# Comment on “Efficacy and Safety of Combined Platelet‐Rich Plasma With Fractional Laser for Adult Patients With Vitiligo: A Systematic Review and Meta‐Analysis of Randomized Controlled Trials”

**DOI:** 10.1111/jocd.70761

**Published:** 2026-03-26

**Authors:** Miloš D. Pavlović

**Affiliations:** ^1^ Faculty of Health Sciences University of Primorska Koper Slovenia

Dear Editor,

1

Value of the succinct, trusted medical information cannot be overestimated for a busy clinician.

In the May issue Feng et al. [1] published their meta‐analysis (MA) assessing efficacy and safety of a combination of platelet‐rich plasma (PRP) and fractional laser (FL) for patients with vitiligo. They concluded that “the combination is a valuable and safe treatment modality for patients with vitiligo, based on its superiority to control groups”. The combination was found to be better than the narrow band UVB phototherapy, a cornerstone treatment of vitiligo. A reduced *no response rate* was also highlighted though this may not be relevant in vitiligo since, to be clinically meaningful to patients, the repigmentation should be above 50% [2].

We reviewed details of both the MA and its primary source studies. Several red flags emerged and we will highlight here only some of them. They illustrate how vulnerable clinicians may be to ostensibly “trusted” information that has the potential to influence clinical practice.

Major flaws in the MA (primary studies' inclusion and bias assessment) are given in Table 1. The modified Jadad scale [5] was used to assess the quality of randomized controlled trials (RCTs) included in the MA. I randomly examined the studies by Afifi and Abdelghani (all primary references are cited in [1]), which were assigned scores of 8 and 5, respectively. These studies would merit only 2–3 points. Although labeled as randomized (+1), the method of randomization was either inappropriate or insufficiently described (“simple randomization,” “randomly categorized”; −1). Blinding was not mentioned (0), withdrawals and dropouts were not described (0), inclusion and exclusion criteria were reported (+1), adverse effects were assessed inappropriately (0), and statistical methods were described (+1). Two studies should not have been included in the analysis at all. The study by Ahlawat included 60 patients but was not a randomized study. The study by Abdel‐Hamid included 40 patients and PRP was applied topically, not intradermally as in all other trials, and hence the procedures cannot be pooled and compared.

**TABLE 1:** Meta‐analysis' pitfalls.

	Observed pitfalls	Comments
Quality of RCT assessment	Modified Jadad score: Afifi et al.: 8 Abdelghani et al.: 5	Actual scores: 2 or 3
Funnel plot construction [3]	Too low number of studies included: 8 Substitution of primary for secondary outcomes	Number of studies qualified for inclusion even lower: 5 Publication bias is known to be strongest for primary outcomes [4]
Random sequence generation	Rated as low risk of bias in Abdelghani et al. and Kadry et al.	Inadequate, with substantial selection bias
Inclusion of inappropriate studies	Ahlawat et al.: not RCT Abdel‐Hamid et al.: topical PRP	Topical PRP application cannot be analyzed together with intradermal injections

Abbreviations: PRP, platelet‐rich plasma; RCT, randomized controlled trial.

Closer examination of the primary studies reveals further concerns (Table 2). In the study by Kadry, it is unlikely that patients would consider a difference between 53% and 57% repigmentation clinically meaningful. In a study by Afify a diameter difference in vitiligo patches of approximately 0.4 mm is simply imperceptible by any human observer. Yet it was factored in the MA as a significant finding favoring FL + PRP.

**TABLE 2:** Selected flaws of primary studies included in the meta‐analysis.

	Studies	Comments
Sample size calculation either not reported or inadequately presented	All	Inadequate sample size calculation leads to underpowered studies, biased and heterogeneous inputs, and uncertain pooled results in MAs
Differences in primary outcomes not significant	Kadry et al.	VACAG 56.83 ± 29.67 for FL + PRP, and 53.05 ± 37.08 for PRP alone CIs (assessed by Welch's test) included zero (no statistically significant difference): the observed difference of 2.79 is consistent with no effect
Primary outcomes differences, though claimed statistically significant, are either implausible or imperceptible	Afify et al.	The mean percentage area reduction was reported as 0.4% (SD 0.2) for PRP and 0.2% (SD 0.1) for FL + PRP. Vitiligo patch surface area before and after FL + PRP 11.9 cm^2^ and 11.7 cm^2^—a diameter difference of approximately 0.4 mm

Abbreviations: CIs, confidence intervals; FL, fractional laser; MA, meta‐analysis; PRP, platelet‐rich plasma; SD, standard deviation; VACAG, Vitiligo Analysis by Computer‐Assisted Grid.

Repigmentation grading scales varied across studies, making direct comparison problematic. In Abdelghani, FL + PRP achieved a mean repigmentation grade of 4.4 (51%–75% repigmentation), whereas PRP alone achieved 1.5 (6%–25%). On the other hand, Kadry reported comparable outcomes (51%–75% repigmentation) for both interventions. Nevertheless, the MA concluded that FL + PRP was superior to PRP, even though the 95% confidence interval crossed zero (−1.07 to 4.17). These physician‐graded improvements were discordant with the absence of objective improvement in VACAG measurements within the same study.

The authors of the MA claimed: “…compared with some traditional treatment methods for vitiligo, such as (…) topical medications or phototherapy, the treatment course of FL combined with PRP is relatively short, and *the effect is more significant…*”.

Although statistical “evidence” may be there, it has no clinical relevance. Publishers must invest in reviewers with expertise in both statistics and clinical science, who can rigorously evaluate submissions and allow publication only of work meeting stringent standards of validity. Meta‐analyses and RCTs exert profound influence on clinical decision‐making and patient care.

## Funding

The author has nothing to report.

## Conflicts of Interest

The author declares no conflicts of interest.

## Linked Articles

This article is linked to Feng et al. papers. To view this article, visit https://doi.org/10.1111/jocd.70245.

## Data Availability

The data that support the findings of this study are available from the corresponding author upon reasonable request.
